# MiCoDe: a web tool for performing microbiome community detection using a Bayesian weighted stochastic block model

**DOI:** 10.1093/bioinformatics/btaf384

**Published:** 2025-07-01

**Authors:** Kevin C Lutz, Shengjie Yang, Tejasv Bedi, Michael L Neugent, Nikita Madhavaram, Bo Yao, Xiaowei Zhan, Nicole J De Nisco, Qiwei Li

**Affiliations:** Department of Health Data Science and Biostatistics, O’Donnell School of Public Health, The University of Texas Southwestern Medical Center, Dallas, TX 75390, United States; Department of Health Data Science and Biostatistics, O’Donnell School of Public Health, The University of Texas Southwestern Medical Center, Dallas, TX 75390, United States; Department of Mathematical Sciences, The University of Texas at Dallas, Richardson, TX 75080, United States; Department of Biological Sciences, The University of Texas at Dallas, Richardson, TX 75080, United States; Department of Health Data Science and Biostatistics, O’Donnell School of Public Health, The University of Texas Southwestern Medical Center, Dallas, TX 75390, United States; Department of Health Data Science and Biostatistics, O’Donnell School of Public Health, The University of Texas Southwestern Medical Center, Dallas, TX 75390, United States; Department of Health Data Science and Biostatistics, O’Donnell School of Public Health, The University of Texas Southwestern Medical Center, Dallas, TX 75390, United States; Department of Biological Sciences, The University of Texas at Dallas, Richardson, TX 75080, United States; Department of Mathematical Sciences, The University of Texas at Dallas, Richardson, TX 75080, United States

## Abstract

**Summary:**

The Microbiome Community Detector (MiCoDe) software is a free user-friendly web tool that is designed to cluster a network of microbial taxa into communities using a Bayesian weighted stochastic block model. MiCoDe also filters the data automatically and accounts for the challenges of microbiome high-throughput sequencing data including high-dimensionality, compositionality, zero inflation, and nonlinearity. While MiCoDe is based on a rigorous statistical unsupervised learning model, our web tool can be easily used by any investigator. Users simply upload a csv file that contains their taxonomic abundance data where rows correspond to samples and columns correspond to taxa. Then, users make a few selections regarding data transformation, network estimation, and the number of communities in order to run the online analysis. If users are unsure of what selections to make, then they can opt for the default settings as these are our recommended settings. In this paper, we discuss the motivation, methodology, implementation, and results of MiCoDe. We also discuss how MiCoDe can be adapted by the user and how it may evolve over time. Our software is a valuable tool for microbiome community detection.

**Availability and implementation:**

MiCoDe is freely available online at https://lce.biohpc.swmed.edu/micode/, does not require installation, and is not browser-specific. Users can also work locally using our R code, which is freely available on GitHub at https://github.com/klutz920/MiCoDe.

## 1 Introduction

The rising interest in advanced microbiome profiling techniques indicates a trend toward more accurate and comprehensive characterization of microbial communities, which is critical for improving our understanding of microbial diversity and function. The ecological interaction of microorganisms in complex communities is fundamental in shaping microbiome function and host health (Antwis *et al.* 2017). Microbiomes harbor intricate associations between member taxa that may be critical to the assembly and function of microbial communities. Identifying and characterizing these associations from high-throughput sequencing data can provide insight into disease progression and susceptibility (Faust *et al.* 2012, Huttenhower *et al.* 2012). For example, Hall *et al.* (2019) found evidence that the human gut microbiome is composed of distinct communities (or clusters) of taxa that interact with each other. Further, similar metagenomic functional properties were observed in each community. Recently, we found evidence that the urinary microbiome of postmenopausal women with recurrent urinary tract infections (rUTI) is composed of distinct communities that harbor taxa known to be associated with dysbiosis (Lutz *et al.* 2025).

*Microbiome community detection* is a fundamental problem of network analysis that aims to uncover the underlying community structure of a microbiome network, which is key to understanding the impact of such a structure on human health. Such an analysis requires a graph, which consists of nodes and edges. In a microbiome network, a node represents a taxon and an existing edge represents a numerical or non-numerical association between any two taxa. Graphs where edges have numerical value such as a correlation coefficient are known as *weighted graphs*. A *community* is a cluster of nodes that are densely connected to each other within that community and sparsely connected to nodes in other communities (Gulbahce and Lehmann 2008).

Microbiome community detection can help characterize taxon-taxon associations and reveal their latent properties, mechanisms, and structures (Jiang *et al.* 2020). Hence, there is an increasing demand for network-based clustering methods to detect communities and investigate associations among microbial taxa (Hall *et al.* 2019), which are essential for advancing our understanding of microbiome dynamics. Numerous methods have emerged to offer new ways to identify and characterize microbial communities. By uncovering community structure, taxa with heretofore unknown functions could be classified into community types implying different disease states that may elucidate their potential function. For example, we recently used a Bayesian stochastic block model (SBM) for an unweighted graph to study the aforementioned community structure of the urinary microbiome in postmenopausal women with rUTI (Lutz *et al.* 2025). These methods can support existing knowledge or help to form new hypotheses about diseases such as rUTI. Moreover, methods that are not tailored to handle the statistical challenges of taxonomic abundance data such as high-dimensionality, zero-inflation, and compositionality are not appropriate for microbiome data analysis (Cullen *et al.* 2020). This oversight can result in information loss and increased bias. Consequently, we developed the Microbiome Community Detector (MiCoDe, pronounced “my code”) software, which is a free web tool designed to perform microbiome community detection using a Bayesian weighted stochastic block model (WSBM) that is tailored to handle the challenges of microbiome data.

Generally, SBMs are probabilistic models that use unsupervised learning to cluster the nodes of a network graph that have heterogeneous connectivity patterns into mutually exclusive blocks (or communities) that have homogeneous connectivity patterns (McDaid *et al.* 2013, Aicher *et al.* 2015, Lee and Wilkinson 2019), providing a natural way to detect communities in a microbiome network (Geng *et al.* 2019). The SBM has been widely utilized in many disciplines including medicine (Li *et al.* 2021), social media (Yu *et al.* 2018), sociology (McDaid *et al.* 2013), political science (Latouche *et al.* 2011), military strategy (Olivella *et al.* 2022), and infrastructure (Yu and Baroud 2020). Applications of the SBM in microbiome community detection include disease development related to the human gut microbiome (Marchesi *et al.* 2016, Hall *et al.* 2019, Bhar *et al.* 2022), pancreatic cancer (Stanley *et al.* 2019), tick-related diseases (Lejal *et al.* 2021), and COVID-19 (Ghavasieh *et al.* 2021). MiCoDe now provides investigators in various disciplines with a fast, reliable, and easy-to-use web tool for performing community detection.

MiCoDe gives the user options to preprocess the taxonomic abundance data using a transformation technique as well as estimate the weighted graph of the microbial network using an association metric to properly correct the data for analysis. The MiCoDe interface is free, user-friendly, requires no installation or programming, and is not browser-specific. To the best of our knowledge, no other web tools for community detection exist. However, NAMData (Aguinaldo *et al.* 2021) was introduced in 2021 as a community detection web tool but is currently unavailable on the web as far as we know. The conference paper for NAMData has only been cited twice since 2021, which could indicate that the software has been unavailable for quite some time. Given this, MiCoDe is likely the only current community detection web tool that is accessible to all investigators including those who may not have expertise in programming or network analysis methods for microbiome data. In the next section and Supplementary Materials, we provide the details about the methodology behind MiCoDe including data preprocessing, estimation of the weighted graph, and the specific SBM that is used to cluster the weighted graph.

## 2 Methods

The goal of MiCoDe is to cluster *p* taxa sequenced from *n* samples into *K* mutually exclusive communities. MiCoDe achieves this goal in three steps. For mathematical and modeling reasons, MiCoDe automatically filters the abundance data prior to these three steps so that there are at least seven non-zero counts for each taxonomic feature. In the first step, MiCoDe applies one of three transformations to preprocess and normalize the microbiome abundance data: (i) scale the abundances in each sample by their library size (i.e. the compositional data or relative abundances), (ii) the centered-log ratio (CLR) transformation (Aitchison 1982, 1984), or (iii) the modified centered-log ratio (MCLR) transformation (Yoon *et al.* 2019). Second, a weighted network graph and its adjacency matrix are constructed from the transformed data using one of three measures of association between pairs of taxa: (i) semi-parametric rank-based (SPR) correlation designed specifically for MCLR-transformed data (Yoon *et al.* 2019), (ii) Spearman correlation, or (iii) Pearson correlation. The weighted graph is assumed to be undirected, fully connected, and has no self-loops. Third, microbiome community detection is performed on the estimated weighted network graph using either a Bayesian WSBM or a weighted stochastic infinite block model (WSIBM).

The parameters of the WSBM estimate block-specific edge weights (e.g. correlation coefficients) and community labels (e.g. cluster or group number). The edge weights are characterized by their mean and variance with normal and inverse-gamma priors assigned to them, respectively. A Dirichlet-multinomial prior is assigned to the discrete community labels. Furthermore, WSBM is a finite block model where *K* is prespecified by the user; whereas, WSIBM is an infinite block model where K→∞. Moreover, WSIBM automatically infers the optimal value of *K via* a truncated stick-breaking construction of the Dirichlet Process (DP) (Ishwaran and James 2001). Thus, the value of *K* does not need to be prespecified or chosen arbitrarily by the user for WSIBM. Model convergence can be assessed by comparing the similarity of multiple independent chains of the MCMC algorithm on the real data. Similarity can be measured using the adjusted Rand index (ARI), which was also used in our previous work for assessing convergence (Lutz *et al.* 2025).

Note that the default settings for preprocessing the data, selecting the correlation metric and number of communities are MCLR, SPR, and Automatic, respectively, because these are our recommended settings especially for when the abundance data are nonlinear and have high zero inflation. MCLR also removes compositionality. Lastly, Bayesian models can handle high-dimensional data.

Details about data preprocessing, weighted graph construction, and the model are available in Sections S1–S3, available as supplementary data at *Bioinformatics* online, respectively, of the supplement. Additional details, including model validation and real data analysis, are available in Bedi *et al.* (2024).

## 3 Implementation

**Figure 1.**
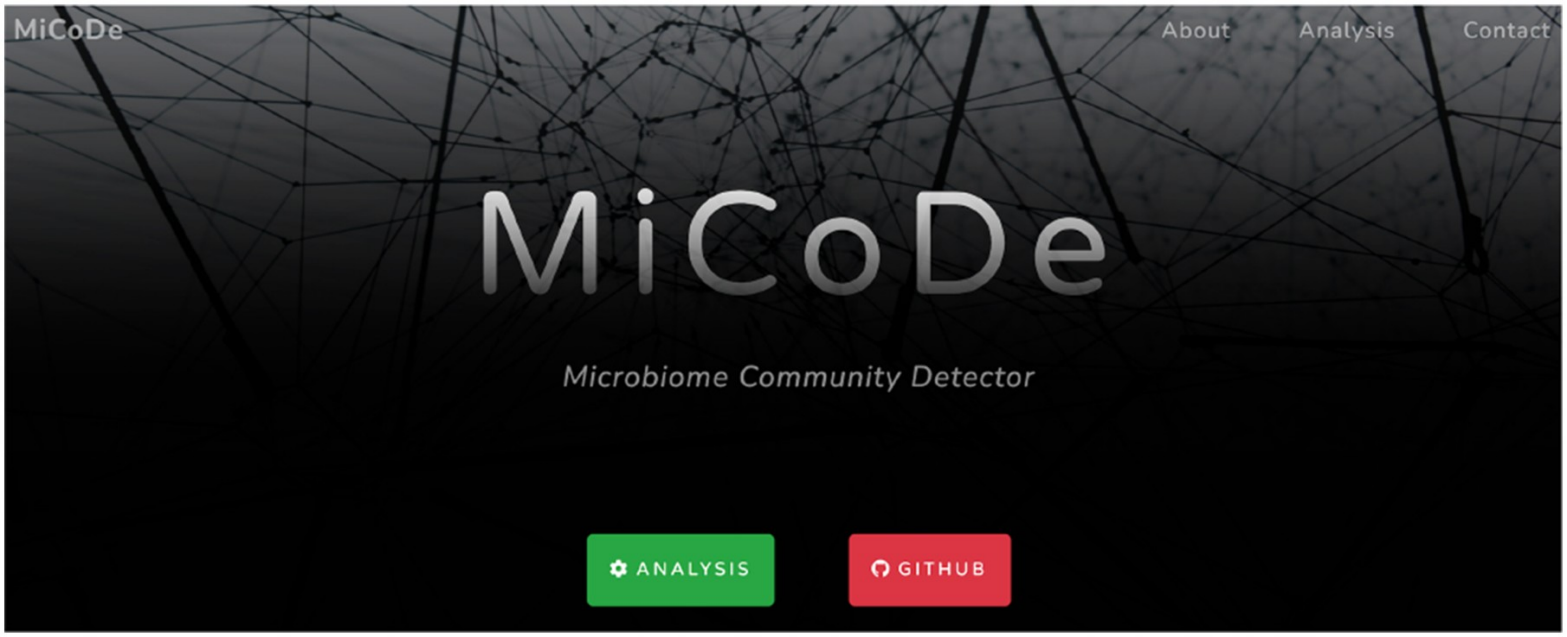
The top of the MiCoDe webpage. Users can either select “Analysis” to scroll down to the analysis section or select “GitHub” to access and download the R and Rcpp source code from our repository to work offline.

**Figure 2.**
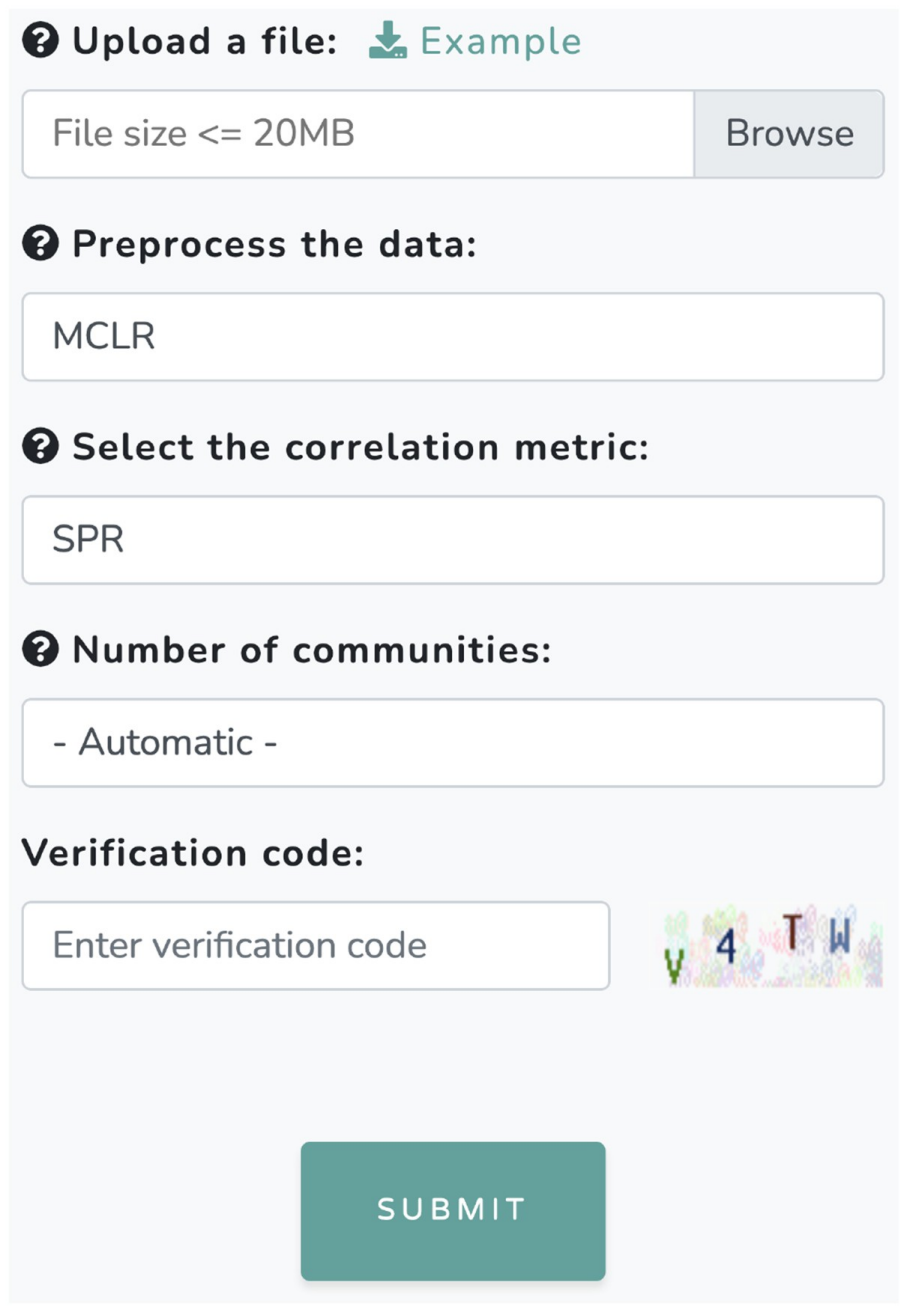
User input for MiCoDe: (i) upload a csv file up to 20 MB in size containing abundance data; (ii) select a transformation (MCLR, CLR, Compositional) to preprocess the data; (iii) select a correlation metric (SPR, Spearman, Pearson) to construct the network graph; (iv) prespecify the number of communities (2–20) to use WSBM or let MiCoDe estimate the optimal number of communities (select “Automatic”) using WSIBM; and (v) enter a verification code (the verification code is randomly generated, so do not use the code in this figure). MCLR, SPR, and Automatic are the default settings. Hovering the mouse over the question marks (?) will reveal more information about each input.

At the top of the MiCoDe interface (Fig. 1), users can click on “Analysis” to quickly scroll down to the online analysis section. Additionally, users can click on “GitHub” to visit our repository at https://github.com/klutz920/MiCoDe to download the R and Rcpp source code to work locally. Users may also tailor the code for their needs (e.g. using another data transformation not offered by MiCoDe or use a different filtering process). When the user scrolls from the top of the interface manually, they will find brief user instructions and data formatting guidelines prior to the analysis section. For online analysis, users must submit the five items shown in Fig. 2. First, users must upload their taxonomic abundance table (not the relative abundances), which must be a .csv file up to 20 MB in size where rows correspond to samples and columns correspond to taxa. For larger files, it is recommended that users run the analysis locally by downloading the source code from our GitHub repository (mentioned earlier). The first row of the table must include the names of the taxa. MiCoDe also has three real example datasets that users can download, view, and then upload for online analysis. Table 1 in Section 4, available as supplementary data at *Bioinformatics* online of the supplement provides information about the example datasets. Second, users must select one of three transformations for data preprocessing: compositional, CLR, or MCLR (default). Third, select a correlation metric for estimating the weighted network graph: SPR (default), Spearman, or Pearson correlation. Fourth, select the number of communities *K* by prespecifying a value from 2 to 20 to use WSBM or by selecting “Automatic” (default) to automatically determine the optimal value of *K via* WSIBM. The choice of a prespecificied value of *K* can be for exploratory purposes or it can based on prior biological knowledge about the number of communities. When there is no prior knowledge, it is recommended to use the “Automatic” setting. Fifth, all users must enter a verification code to confirm their identity. Then, click “Submit” to run the analysis, which will take only a few minutes depending on file size. In the next section, we discuss the results of MiCoDe: a dynamic and visual representation of the estimated communities.

## 4 Results

Once online analysis has finished, MiCoDe provides results that can be saved online and downloaded. To illustrate the results that users will observe when using MiCoDe, we analyzed one of the example data files (example1.csv) and have provided select results in the figures below and in the supplement for demonstration purposes. These data were preprocessed using MCLR, SPR was selected for the correlation metric, and K=5 was selected for the number of communities. Thus, the WSBM was utilized for this analysis.

**Figure 3.**
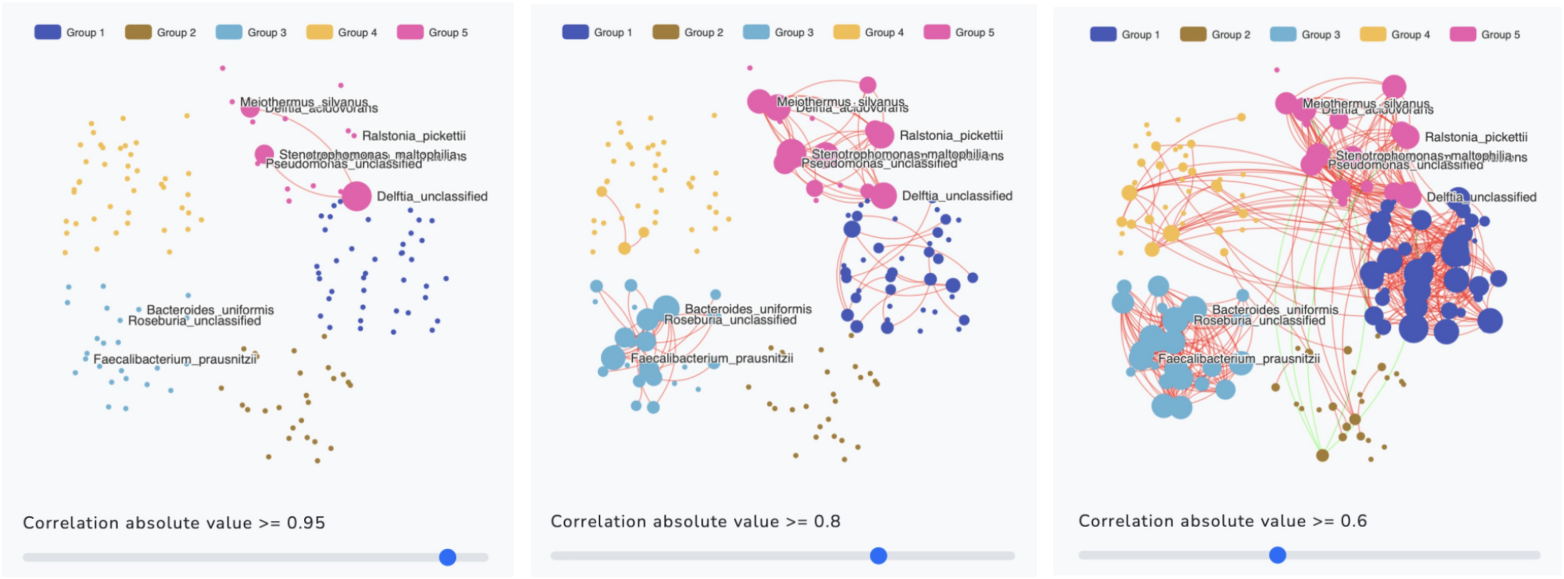
The network graph with the five communities (groups) estimated by WSBM for the example1.csv dataset with absolute correlation thresholds of 0.95, 0.8, and 0.6. A node corresponds to a taxon. An edge represents that the correlation between two taxa is greater than the given threshold. Node size increases as the number of edges connected to that node increases. The number of edges increases as the correlation threshold decreases.

MiCoDe displays a visual network of nodes (i.e. taxa) and weighted edges (i.e. correlation coefficient of two taxa), which illustrates the interactions between taxa that have been clustered into *K* distinct communities. The output is also an interactive network (see Fig. 3). Users can adjust the absolute correlation threshold to any value between 0.3 and 1, prompting edges to appear between any two taxa if the absolute value of the correlation between them is greater than the threshold. The network becomes more connected by lowering the threshold and more sparse by increasing the threshold. We intentionally exclude weaker correlations (<0.3) to minimize clutter in the plot. However, these weaker correlations are included in the results that users can download. Red and green edges indicate positive and negative correlation, respectively, between two taxa.

**Figure 4.**
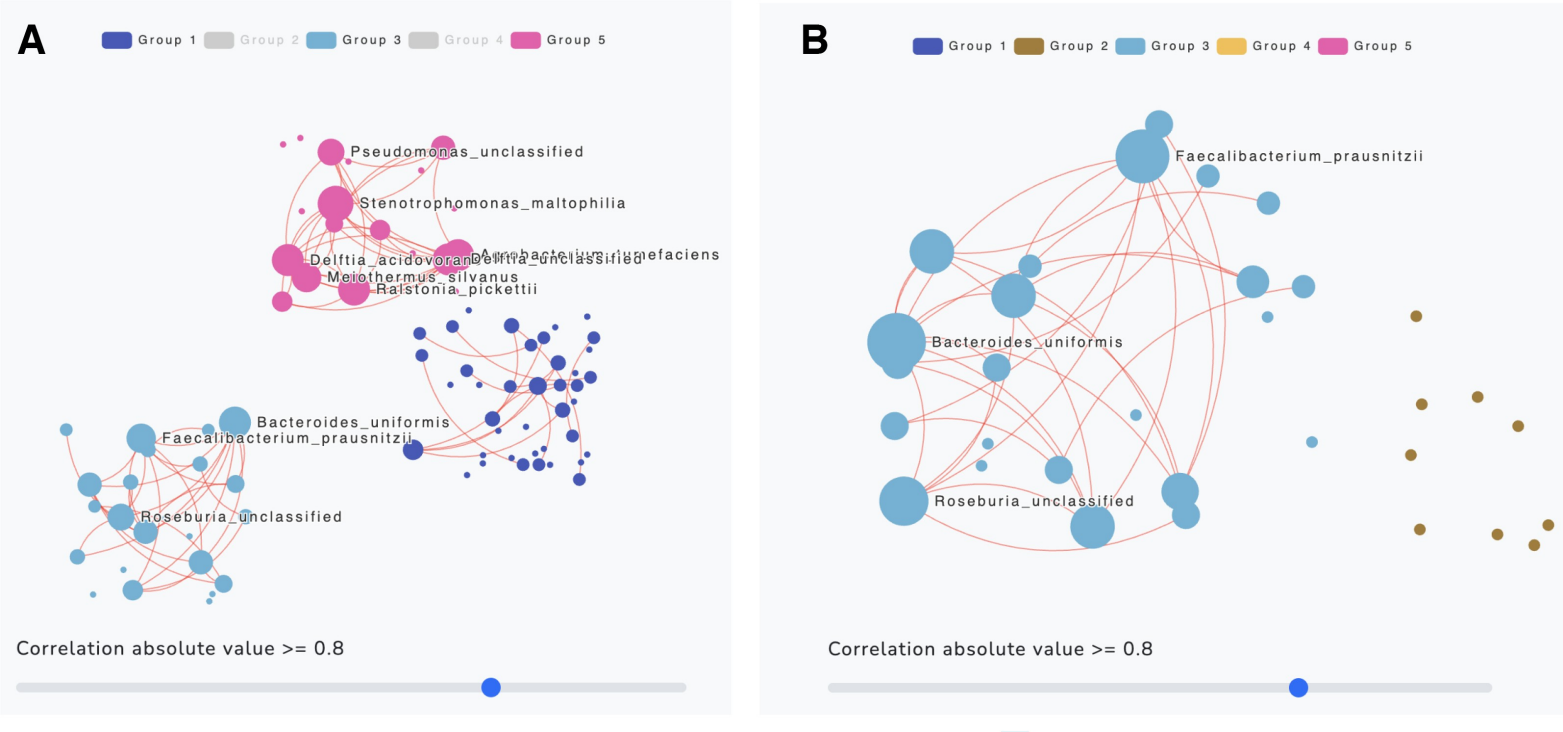
The network graph with the five communities (groups) estimated by WSBM for the example1.csv dataset with absolute correlation threshold 0.8. (A) Groups 2 and 4 are removed by clicking on their names in the legend at the top of the plot. Notice in the legend how both of their names are light gray; (B) A zoomed-in view of Group 3.

**Figure 5.**
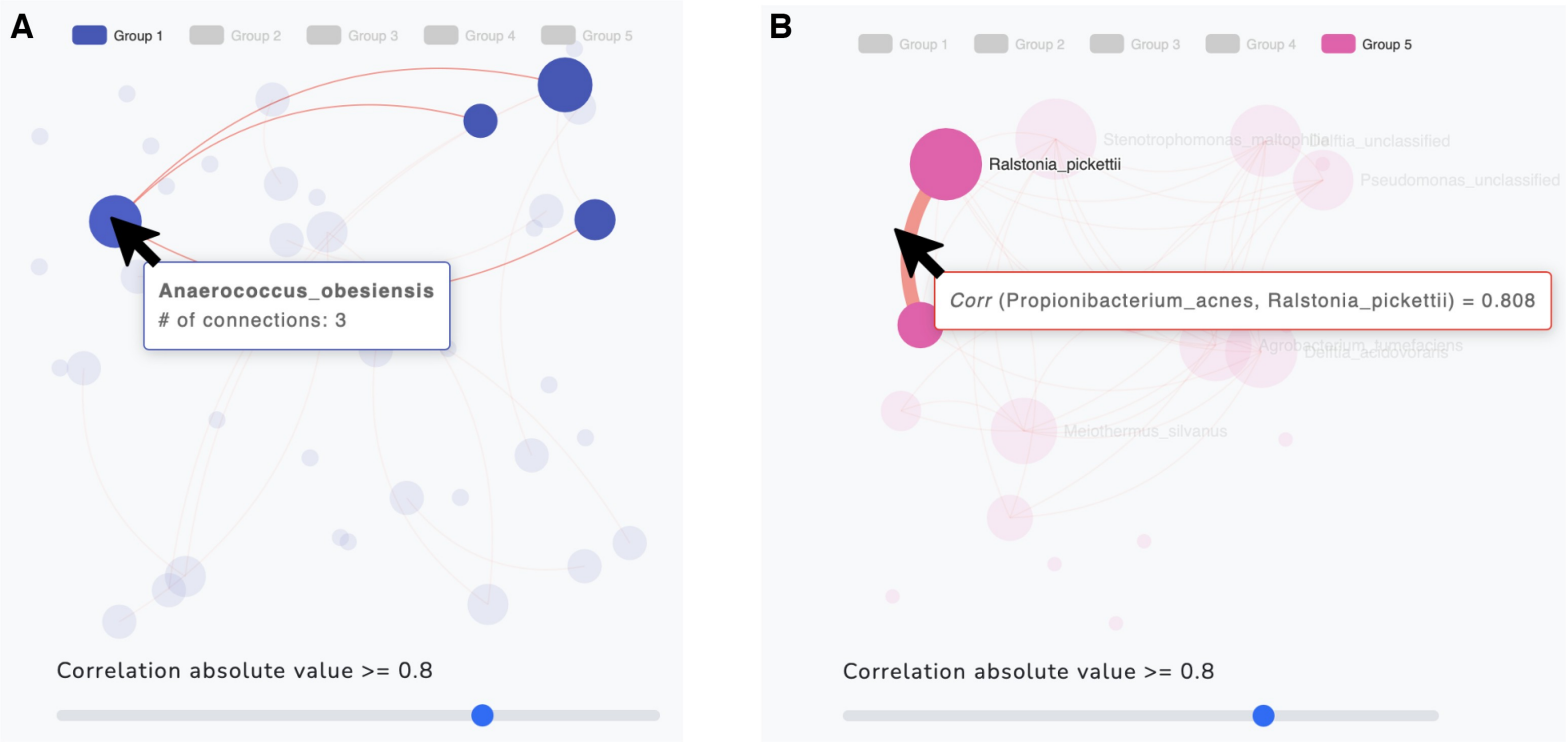
The network graph with the five communities (groups) estimated by WSBM for the example1.csv dataset with absolute correlation threshold 0.8. (A) By hovering the mouse (black arrow) over a node in Group 1, the name of the taxon (*Anaerococcus obesiensis*) and the number of connecting edges to other taxa (=3) appear in a pop-up box; (B) By hovering the mouse (black arrow) over an existing edge in Group 5, the SPR correlation coefficient (prespecified by user) of the two taxa appears in a pop-up box. The correlation is positive because the edge is red. Here, the SPR correlation coefficient of *Propionibacterium acnes* and *Ralstonia pickettii* is 0.808.

Nodes in the same community are grouped according to color. This allows users to identify taxa that belong to the same community. Additionally, the user can visualize the interaction of taxa in the overall network as well as within and between communities. To filter out a community from the visualization, click on the group name in the legend and click again to undo. For example, Fig. 4A illustrates the removal of Groups 2 and 4. Users can also zoom in and out for closer inspection of the results (Fig. 4B). A node’s size is reflective of its number of connecting edges given the set threshold. When hovering over a specific node, the name of the taxonomic feature (based on user specification) and the number of connecting edges will appear in a pop-up box (Fig. 5A). Similarly, hovering over an edge reveals the names of two corresponding taxa and their estimated correlation coefficient in a pop-up box (Fig. 5B).

All results are saved online *via* the provided URL (found above the plot legend) allowing users to revisit or share current analyses without having to rerun the analysis. The reader can access the saved results of this demonstration at https://lce.biohpc.swmed.edu/micode/index.php? jobid=demo. MiCoDe also allows users to download the correlation matrix and clustering result as two separate .csv files named corr_matrix.csv and cluster_result.csv, respectively. The correlation matrix (Fig 1, available as supplementary data at *Bioinformatics* online, Section 4, available as supplementary data at *Bioinformatics* online) is symmetric about the main diagonal and contains the correlation coefficients of all pairs of taxa. This matrix is also the adjacency matrix of the weighted network graph. Because our weighted graph assumes no self-loops, the correlation coefficients along the main diagonal are all zeros. The entire matrix is aggregated in Fig. 2, available as supplementary data at *Bioinformatics* online with statistical summaries of the correlation coefficients of all edges within and between the five communities. The clustering result (Fig. 3, available as supplementary data at *Bioinformatics* online, Section 4, available as supplementary data at *Bioinformatics* online) has two columns: the name (column name: node_name) and estimated community (column name: cluster_index) of each taxon. The estimated community of a taxon is indexed as a discrete number. The taxa are listed in alphabetical order. It may be helpful to sort this file by community index to inspect the taxa assigned to the same community to infer any biological meaning.

The results of this analysis (in particular see Fig. 2, available as supplementary data at *Bioinformatics* online) are biologically coherent in several ways that align with existing evidence in the literature (Shipitsyna *et al.* 2013, Thomas-White *et al.* 2018, Ceccarani *et al.* 2019, Vaughan *et al.* 2021, Neugent *et al.* 2022). Community 1 reflects the known co-occurrence of *Gardnerella vaginalis* and *Atopobium vaginae* in dysbiotic urinary and vaginal microbiomes. Community 2 includes many *Lactobacillus* species with low internal correlation, which is consistent with their ecology. Communities dominated by *Lactobacillus* are typically mono-dominant, with few species co-occurring at high abundance. The largely negative associations between communities 1 and 2 align with the well-known antagonism between taxa associated with bacterial vaginosis and *Lactobacillus*-dominated states. Community 4 contains gut-associated taxa commonly detected in urinary microbiomes, consistent with either contamination or secondary colonization. Communities 3 and 5 include less characterized taxa often seen in dysbiosis, and their strong within and between community correlations reflect a tendency to co-occur.

## 5 Discussion and conclusion

In summary, MiCoDe is a freely available web tool that performs community detection on microbiome abundance data that can be uploaded as .csv file for analysis. MiCoDe will transform the data to account for the challenges of the microbiome data, estimate the weighted graph to construct the microbial network, and then utilizes a robust Bayesian weighted stochastic block model to cluster the estimated microbial network into communities of taxa. Users can specify the number of communities (using the WSBM) or let the model determine the best number (using the WSIBM). The results of MiCoDe include an interactive plot with several metrics that can be saved online and downloaded onto a computer. MiCoDe is a new powerful tool for more easily facilitating advanced microbiome studies by uncovering community structure in microbial networks using fast and reliable methodology. MiCoDe is available at https://lce.biohpc.swmed.edu/micode/. The R and Rcpp source code for MiCoDe is also freely available on GitHub (https://github.com/klutz920/MiCoDe) if users wish to adjust the code for their needs or run an offline analysis. Users should cite this paper and/or the website when publishing any results generated by MiCoDe.

MiCoDe has the potential to evolve over time. Currently, MiCoDe allows the user to choose from three transformation techniques and three association metrics. Options may increase over time as newer validated methods become available. If requested, we would also consider including other techniques and metrics that are appropriate for microbiome data analysis. MiCoDe currently does not have the option to leverage covariates; however, the model can be adjusted to include them. Lastly, we invite users to email us to make recommendations for improving MiCoDe.

## Supplementary Material

btaf384_Supplementary_Data

## Data Availability

Source code for MiCoDe is available through Zenodo (https://doi.org/10.5281/zenodo.15345743) or our GitHub repository (https://github.com/klutz920/MiCoDe).
