# Supplementary material for: MiCoDe: a web tool for performing microbiome community detection using a Bayesian weighted stochastic block model
Source: Bioinformatics. 2025 Jul 1;41(7):btaf384. doi: 10.1093/bioinformatics/btaf384 (PMC12263104; doi:10.1093/bioinformatics/btaf384)
Supplement: btaf384_Supplementary_Data [file btaf384_supplementary_data.pdf]

Supplementary Materials for "MiCoDe: A web tool for performing microbiome community detection using a Bayesian weighted stochastic block model"

Kevin C. Lutz*[1], Shengjie Yang[1], Tejasv Bedi[2], Michael L. Neugent[3], Nikita Madhavaram[1], Bo Yao[1], Xiaowei Zhan[1], Nicole J. De Nisco[3], and Qiwei Li†[2]

[1]O'Donnell School of Public Health, The University of Texas Southwestern Medical Center
[2]Department of Mathematical Sciences, The University of Texas at Dallas
[3]Department of Biological Sciences, The University of Texas at Dallas

This document provides supplementary material including a summary of the details about the types data transformations available in MiCoDe, the construction of the weighted graph for the taxa, the weighted stochastic block model (WSBM), the weighted stochastic infinite block model (WSIBM), and additional tables and figures. The full details about the model including model validation can be found in Bedi et al. (2024).

S1 Data Transformations

In this section, we provide the details of the three transformations that can be applied to the abundance data when using MiCoDe. In each transformation, x_{ij} represents the count for taxon j in sample i where $i = 1, \dots, n$ and $j = 1, \dots, p$.

1. **Compositional data (i.e., relative taxonomic abundance data).** Let y_{ij} denote the relative abundance for taxon j in sample i where

$$y_{ij} = \frac{x_{ij}}{s_i}$$

   and s_i is the library size of sample i.

2. **Centered-log ratio (CLR).** Aitchison (1982, 1984) proposed the CLR transformation to remove the compositional constraint from the relative abundances. This transformation first adds a pseudo-value to each x_{ij} and then re-normalizes the data to maintain compositionality. Let $\tilde{y}_{ij}$ denote the re-normalized relative abundances. Then, the CLR transformation on $\tilde{y}_{ij}$ is given by

$$f(\tilde{y}_{ij}) \quad = \quad \log\left[\frac{\tilde{y}_{ij}}{g(\tilde{\boldsymbol{y}}_i)}\right]$$

   where $g(\tilde{\boldsymbol{y}}_i) = (\prod_{j=1}^{p} \tilde{y}_{ij})^{1/p}$ is the geometric mean of the i-th sample.

*Corresponding author: Kevin.Lutz@UTSouthwestern.edu
†Corresponding author: Qiwei.Li@UTDallas.edu

3. **Modified centered-log ratio (MCLR).** The CLR transformation has several disadvantages. CLR masks the zeros in the data by adding a pseudo-value to the abundances; thus, it is not able to account for zero inflation in the data. As a consequence, CLR may treat zeros and non-zeros equally by adding a pseudo-value to the abundance data. Lastly, choice of pseudo-value is arbitrary, which may reduce interpretability and increase bias. Yoon et al. (2019) proposed the MCLR transformation, which preserves the zeros in the data by applying the CLR transformation to only nonzero relative abundances. Let $\boldsymbol{y}_i = (y_{i1}, \ldots, y_{ip})^\top$ denote p-dimensional vector of relative abundances belonging to sample i. The MCLR transformation on $\boldsymbol{y}_i$ is given by

$$f(\boldsymbol{y}_i) \quad = \quad \left[0, \ldots, 0, \log\left(\frac{y_{i(j^*+1)}}{\tilde{g}(\boldsymbol{y}_i)}\right) + \epsilon_i, \ldots, \log\left(\frac{y_{ip}}{\tilde{g}(\boldsymbol{y}_i)}\right) + \epsilon_i\right]$$

where j^* is the number of zero relative abundances and $\tilde{g}(\boldsymbol{y}_i) = (\prod_{j=j^*+1}^{p} y_{ij})^{1/(p-j^*)}$ is the geometric mean of the $p - j^*$ non-zero relative abundances with the adjustment factor $\epsilon_i = |\min_{j:y_{ij}\neq 0} \log(y_{ij}/\tilde{g}(\boldsymbol{y}_i))| + 1$ as suggested by Yoon et al. (2019) who imposed a shift to ensure all non-zero transformed values are strictly positive.

S2 Weighted Graph Construction for Taxonomic Abundance Data

Here, we give details on how to construct the weighted graph from the transformed taxonomic abundances. Each taxon is represented by a node. The weighted graph is assumed to represent a fully connected, undirected network with no self-loops. This means that any two taxa j and j' where $j \neq j'$ are connected by a weighted edge, which is denoted by $R_{jj'}$ and is calculated from the transformed abundance data using a pairwise measure of association. This will obtain the adjacency matrix $\boldsymbol{R} = [R_{ij}] \in [-1, 1]^{p\times p}$ where $R_{jj'} = R_{j'j}$ when $j \neq j'$ and $R_{jj'} = 0$ when $j = j'$. MiCoDe allows users to select Pearson, Spearman, or SPR correlation to estimate the weight of each edge of the graph. It is important to know that both Pearson and Spearman do not account for zero inflation. In addition, Pearson does not account for nonlinearity in the data. SPR accounts for both nonlinearity and zero inflation. Finally, we apply the Fisher transformation $\mathcal{F} : [-1, 1] \longrightarrow \mathbb{R}$ on each $R_{jj'}$ to obtain a weight matrix $\boldsymbol{W} = [W_{jj'}] \in \mathbb{R}^{p\times p}$ where

$$W_{jj'} \quad = \quad \mathcal{F}(R_{jj'}) \quad = \quad \frac{1}{2}\ln\left(\frac{1 + R_{jj'}}{1 - R_{jj'}}\right).$$

The final transformation step maps the correlations to the real number line, which is necessary to satisfy the distributional assumptions of the Weighted Stochastic Block Model (WSBM) and Weighted Stochastic Infinite Block Model (WSIBM). Additionally, the Fisher transformation of the correlations results in a bell-shaped distribution.

S3 Model

S3.1 Finite Block Model

In this section, we briefly discuss the details of WSBM, which is a finite block model that clusters p nodes (i.e., taxa) into a fixed number of mutually exclusive communities (or clusters). The fixed number of communities is denoted as K. The three model parameters that are inferred are $\boldsymbol{z}, \boldsymbol{\Theta}$, and $\boldsymbol{\tau}$, which are described below.

- **Community allocation vector, $\boldsymbol{z}$.** The discrete-valued vector $\boldsymbol{z} = [z_j] \in \mathbb{N}_+^{1\times p}$ gives the community membership of each node where $z_j = k$ for $k = 1, \dots, K$. The expression $n_k = \sum_{j=1}^{p} I(z_j = k)$ gives the total number of vertices belonging to community k and $I(\cdot)$ is the indicator function. Next, each block is denoted as $\boldsymbol{W}^{[l,q]}$ and is a sub-matrix of $\boldsymbol{W}$. Specifically, $\boldsymbol{W}^{[l,q]} = \{W_{jj'} : z_j = l, z_{j'} = q\}$ for all pairs $(l,q) = 1, \dots, K$. Here, the diagonal blocks $\boldsymbol{W}^{[l,l]}$ for $l = 1, \dots, K$ have within-community interactions (i.e., any two taxa belonging to the same community). While, the off-diagonal blocks $\boldsymbol{W}^{[l,q]}$ for $l \neq q$ have between-community interactions (i.e., any two taxa belonging to two different communities). We further assume $\boldsymbol{W}^{[l,q]} = \boldsymbol{W}^{[q,l]}$, resulting in $K(K+1)/2$ blocks in total.

- **Mean and variance parameters of block-specific edge weights, $\boldsymbol{\Theta}$..** Edge weights in block are characterized by their own mean and variance, which we denoted as $\boldsymbol{\Theta} = \{\mu_{l,q}, \sigma^2_{l,q}\}$. We specify the normal (N) and inverse gamma (IG) priors as follows: $\mu_{l,q}|\sigma^2_{l,q} \sim N(\mu_0, \sigma^2_{l,q}/n_0)$ and $\sigma^2_{l,q} \sim \text{IG}(\nu_0/2, SS_0/2)$ with fixed hyperparameters $\mu_0 \in \mathbb{R}, n_0 > 0, \nu_0 > 0$, and $SS_0 > 0$ prespecified for non-informative settings. Then, we can derive the posterior densities of block means and variances $\boldsymbol{\Theta}_{l,q}$ for all $(l,q) = 1, \dots, K$. Due to conjugacy, we obtain the posterior densities of $\boldsymbol{\Theta}_{l,q}$ given $\boldsymbol{z}$ by

$$\begin{aligned} \pi(\mu_{l,q}|\boldsymbol{z}, \sigma^2_{l,q}, \boldsymbol{W}^{[l,q]}) &\propto N(\mu_{l,q},\ \sigma^2_{l,q}/n_{l,q}) \\ \pi(\sigma^2_{l,q}|\boldsymbol{z}, \boldsymbol{W}^{[l,q]}) &\propto \text{IG}(\nu_{l,q}/2,\ SS_{l,q}/2) \end{aligned}$$

  where total number of edges $N_{l,q}$ and sample mean $\overline{W}_{l,q}$ for block $\boldsymbol{W}^{[l,q]}$ are defined by

$$N_{l,q} = \begin{cases} \binom{n_l}{2}, & \text{if } l = q \\ n_l n_q, & \text{otherwise} \end{cases} \quad \text{and} \quad \overline{W}_{l,q} = \frac{\sum_{(j,j')\in\mathcal{S}_{l,q}} W_{jj'}}{N_{l,q}},$$

  respectively. Subsequently, the posterior parameters are $n_{l,q} = N_{l,q} + n_0$, $\nu_{l,q} = N_{l,q} + \nu_0$, $\mu_{l,q} = \frac{N_{l,q}\overline{W}_{l,q} + n_0\mu_0}{N_{l,q}+n_0}$ and $SS_{l,q} = SS_0 + \sum_{(j,j')\in\mathcal{S}_{l,q}} (W_{jj'} - \overline{W}_{l,q})^2 + \frac{n_0 N_{l,q}}{N_{l,q}+n_0}(\overline{W}_{l,q} - \mu_0)^2$.

- **Community allocation probability, $\boldsymbol{\tau}$.** We assign a multinomial (Mult) prior on $\boldsymbol{z}$, which is written as $\boldsymbol{z}|\boldsymbol{\tau} \sim \text{Mult}(1; \tau_1, \dots, \tau_K)$. This allows us to sample a community label for each node given the probability of membership to each community τ_k. We assume $\boldsymbol{\tau}$ is random. So, we place a Dirichlet (Dir) hyperprior on the probability vector $\boldsymbol{\tau}|\boldsymbol{\eta} \sim \text{Dir}(\eta_1, \dots, \eta_K)$ where $\boldsymbol{\eta}$ is fixed. The posterior density of z_j is

$$\pi(z_j = l|\boldsymbol{\Theta}, \boldsymbol{W}, \boldsymbol{\tau}) \quad \propto \quad \left[\prod_{q=1}^{K} f(\boldsymbol{W}^{[l,q]}|z_j = l, z_{j'} = q, \boldsymbol{\Theta}_{l,q})\right] \pi(z_j = l|\boldsymbol{\tau}).$$

  We then normalize the posterior densities of z_j for each node to estimate the probabilities of community membership for block l by

$$p_{jl} = p(z_j = l|\boldsymbol{\Theta}, \boldsymbol{W}, \boldsymbol{\tau}) = \frac{\pi(z_j = l|\boldsymbol{\Theta}, \boldsymbol{W}, \boldsymbol{\tau})}{\sum_{q=1}^{K} \pi(z_j = q|\boldsymbol{\Theta}, \boldsymbol{W}, \boldsymbol{\tau})}.$$

  Last, we sample the community label z_j from a multinomial distribution given by $z_j|\boldsymbol{\Theta}, \boldsymbol{W}, \boldsymbol{\tau} \sim \text{Mult}(1, p_{j1}, \dots, p_{jK})$ and also update $\boldsymbol{\tau}$ *via* Dirichlet-multinomial conjugacy given by $\boldsymbol{\tau}|\boldsymbol{z}, \boldsymbol{\eta} \sim \text{Dir}(n_1 + \eta_1, \dots, n_K + \eta_K)$.

Finally, the parameters $\boldsymbol{z}$, $\boldsymbol{\Theta}$, and $\boldsymbol{\tau}$ can be estimated for a fixed number of communities K *via* a blocked Gibbs sampler that updates these parameters successively in blocks.

S3.2 Infinite Block Model

We further extend our model to the WSIBM that automatically infers K *via* a truncated stick-breaking construction of the Dirichlet Process (DP) (Ishwaran and James, 2001). While this model assumes that K is infinite, the value of K is determined by the number of non-empty communities since the number of nodes p is assumed to be finite. The three parameters of interest for WSIBM are $\boldsymbol{\rho}$, $\boldsymbol{z}$, and $\boldsymbol{\Theta}$. In this model, $\boldsymbol{\Theta}$ is updated the same way as in WSBM. The parameter $\boldsymbol{\rho}$ contains the community weights constructed *via* the stick-breaking process. The distribution of $\boldsymbol{\rho}$ is called the Griffiths-Engen-McCloskey (GEM) distribution (Ewens, 1990), which is a special case of the DP where a stick of unit length is successively broken into smaller pieces. The length of each piece represents the probability of the k-th community, which is denoted as ρ_k, and is defined by

$$\rho_k = V_k \prod_{s<k} (1 - V_s) \text{ for } k = 2, \dots, K_{\max} - 1 \tag{1}$$

where $V_k \sim \text{Beta}(1, \alpha)$ and Equation (1) together form the GEM distribution. Truncation is imposed by assuming $V_{K_{\max}} = 1$ and $\rho_{K_{\max}} = 1 - \sum_{s=1}^{K_{\max}-1} \rho_s$. This process only allows a finite number of communities and has exponentially decreasing class weights as the number of communities increases. One possible choice is to let $K_{\max} = p$. To update each $\rho_k \in \boldsymbol{\rho}$, we sample V_k^* from the conditional posterior density

$$V_k^* | \boldsymbol{z} \quad \sim \quad \text{Beta}\left(1 + n_k,\ \alpha + \sum_{l=k+1}^{K_{\max}} n_l\right)$$

where $n_k = \sum_{j=1}^{n} I(z_j = k)$. After obtaining the estimated probability $\boldsymbol{\rho}$, we calculate the normalized probability of node j belonging to community k using $p_{jk} = \frac{\rho_k \pi(z_j = k | \boldsymbol{\Theta}, \boldsymbol{W})}{\sum_{l=1}^{K_{\max}} \rho_l \pi(z_j = l | \boldsymbol{\Theta}, \boldsymbol{W})}$. Then, we can sample z_j from $z_j | \boldsymbol{\rho}, \boldsymbol{\Theta}, \boldsymbol{W} \sim \text{Mult}(1, p_{j1}, \dots, p_{jK_{\max}})$. All three parameters can be updated using a blocked Gibbs sampler.

S4 Supplemental Tables and Figures

Table S1: The names, number of rows or samples n, number of columns or taxa p, the file sizes, file size relative to the 20 MB file size limit, their corresponding microbiome, and source for the three example data sets available for download on MiCoDe. For these three data sets, notice how small each file size is relative to the 20 MB limit. This indicates that MiCoDe can accommodate very large abundance tables.

File Name	Samples,n	Taxa, p	File Size	% of 20 MB Limit	Microbiome	Source
example1.csv	86	383	88 KB	0.44%	Urinary	Neugent et al. (2022)
example2.csv	613	199	311 KB	1.56%	Gut	Goodrich et al. (2014)
example3.csv	69	90	23 KB	0.001%	Intestinal	Scher et al. (2013)

	Acinetobacter_ unclassified	Actinobaculum_ massiliense	Actinobaculum_ schaalii	Actinomyces_ europaeus	Actinomyces_ neuil	Actinomyces_ turicensis	Aerococcus_ urinae	Agrobacterium_ tumefaciens	Agrobacterium_ unclassified	Akkermansia_ muciniphila	Alloscardovia_ omnicolens	Anaerococcus_ hydrogenalis
Acinetobacter_ unclassified	0	-0.1355	0.055	-0.1474	0.2602	-0.0808	0.1693	0.6488	0.5727	-0.4167	0.0365	-0.1586
Actinobaculum_ massiliense	-0.1355	0	0.0967	0.2539	0.1677	0.2942	0.1042	-0.1733	-0.198	0.1007	0.0582	0.2451
Actinobaculum_ schaalii	0.055	0.0967	0	0.4549	0.3358	0.3771	0.7267	0.0282	-0.0393	-0.0928	0.0994	0.2037
Actinomyces_ europaeus	-0.1474	0.2539	0.4549	0	0.3671	0.2819	0.3422	0.2016	0.0678	0.2777	0.0177	0.5851
Actinomyces_ neuil	0.2602	0.1677	0.3358	0.3671	0	0.1256	0.1647	0.5151	0.3864	-0.1196	0.1675	0.6896
Actinomyces_ turicensis	-0.0808	0.2942	0.3771	0.2819	0.1256	0	0.3003	-0.1129	0.0041	-0.0518	0.2921	0.1522
Aerococcus_ urinae	0.1693	0.1042	0.7267	0.3422	0.1647	0.3003	0	0.0409	-0.0024	-0.2172	0.064	0.0661
Agrobacterium_t umefaciens	0.6488	-0.1733	0.0282	0.2016	0.5151	-0.1129	0.0409	0	0.8306	-0.1931	0.0213	0.2426
Agrobacterium_ unclassified	0.5727	-0.198	-0.0393	0.0678	0.3864	0.0041	-0.0024	0.8306	0	-0.4197	0.2665	0.1171
Akkermansia_ muciniphila	-0.4167	0.1007	-0.0928	0.2777	-0.1196	-0.0518	-0.2172	-0.1931	-0.4197	0	-0.3422	0.2998
Alloscardovia_ omnicolens	0.0365	0.0582	0.0994	-0.0177	0.1675	0.2921	0.064	0.0213	0.2665	-0.3422	0	0.0141
Anaerococcus_ hydrogenalis	-0.1586	0.2451	0.2037	0.5851	0.6896	0.1522	0.0661	0.2426	0.1171	0.2998	0.0141	0

Figure S1: A sample of the symmetric correlation matrix from analysis on the `example1.csv` data set mentioned in the Results section of the manuscript. This is the adjacency matrix of the estimated microbiome network. Correlations are zero along the main diagonal because our algorithm assumes no self-loops in the graph. When users download their results, the file named `corr_matrix.csv` contains the full correlation matrix with all taxa from the data set.

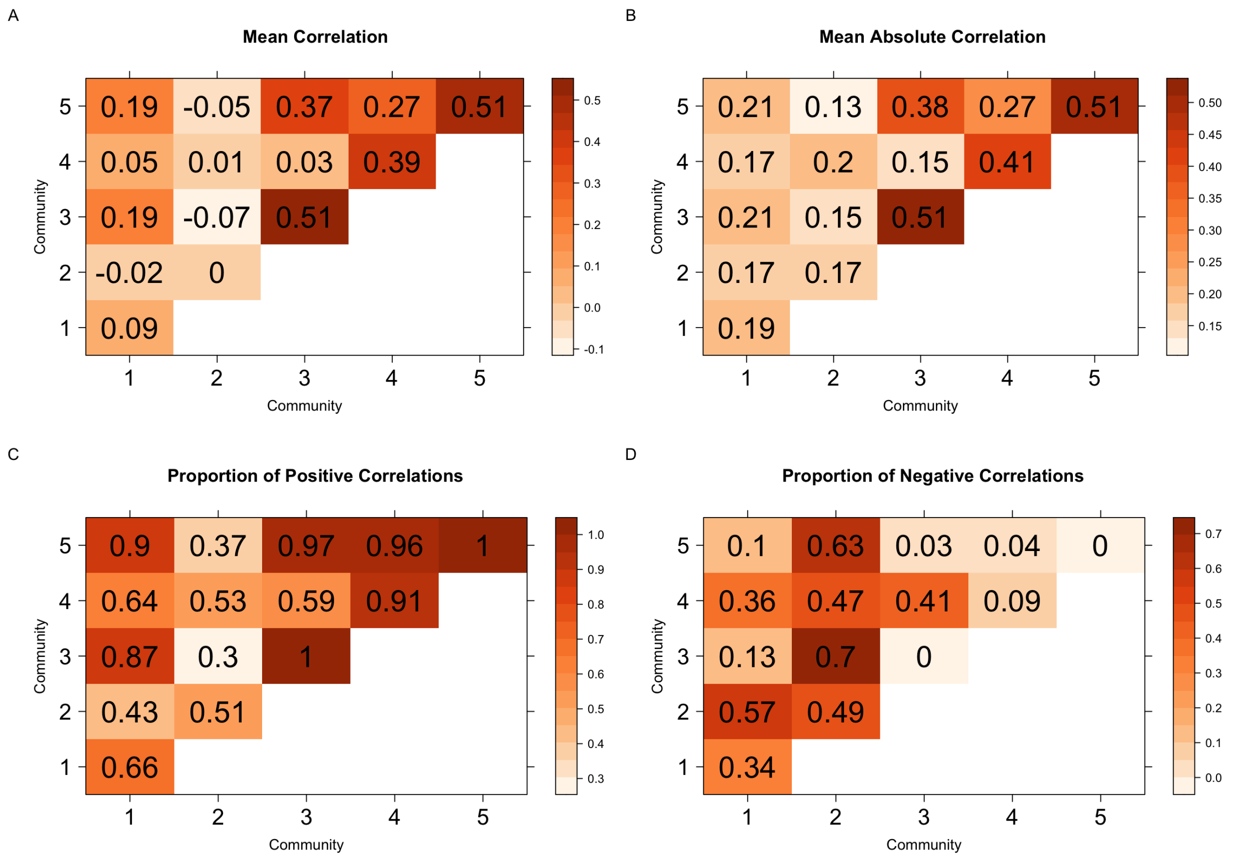

Figure S2: Level plots with statistical summaries of the symmetric correlation matrix (Figure S1) from the real data analysis. Diagonal values (bottom-left to top-right) correspond to within-community summaries and off-diagonal values correspond to between-community summaries. Plot A: Mean correlation of all edges within and between communities; Plot B: Mean absolute value of correlation of all edges within and between communities; Plot C: the proportion of edges within and between communities having positive correlation coefficients; Plot D: the proportion of edges within and between communities having negative correlation coefficients. Plots were generated from the results contained in `corr_matrix.csv` and `cluster_result.csv` using the `levelplot` function from the `lattice` library in R. To illustrate, the mean correlation within community 5 was 0.51 and all correlation coefficients were positive (none were negative), so the mean absolute correlation is also 0.51. The mean correlation between communities 2 and 3 was -0.07, the mean absolute correlation was 0.15, and 70% of the correlation coefficients were negative.

node_name	cluster_index
Acinetobacter_unclassified	2
Actinobaculum_massiliense	1
Actinobaculum_schaalii	3
Actinomyces_europaeus	5
Actinomyces_neuii	5
Actinomyces_turicensis	3
Aerococcus_urinae	3
Agrobacterium_tumefaciens	4
Agrobacterium_unclassified	4
Akkermansia_muciniphila	4
Alloscardovia_omnicolens	1
Anaerococcus_hydrogenalis	5
Anaerococcus_lactolyticus	3
Anaerococcus_obesiensis	5
Anaerococcus_prevotii	5
Anaerococcus_vaginalis	5
Anaeroglobus_geminatus	3
Atopobium_parvulum	1
Atopobium_vaginae	1
Bacteroides_fragilis	4
Bacteroides_uniformis	4
Bacteroides_vulgatus	4
Bifidobacterium_bifidum	1
Bifidobacterium_breve	1
Bifidobacterium_dentium	2
Bifidobacterium_longum	2
BK_polyomavirus	2
Bradyrhizobium_sp_DFCI_1	4
Brevibacterium_massiliense	1
Burkholderia_unclassified	4
Campylobacter_hominis	1

Figure S3: A sample of the first 32 rows of the clustering result for the analysis on the `example1.csv` data set mentioned in the Results section of the main manuscript. The first column (`node_name`) gives the names of the first 23 taxa in alphabetical order. The second column (`cluster_index`) gives the community or group number for each taxon. When users download this result, the file named `cluster_result.csv` is provided and will contain the names and communities of all taxa in the data set. It may be helpful to sort this file in order by `cluster_index` to inspect which taxa are clustered together.

References

Aitchison, J. (1982). The statistical analysis of compositional data. *Journal of the Royal Statistical Society: Series B (Methodological)*, 44(2):139–160.

Aitchison, J. (1984). The statistical analysis of geochemical compositions. *Journal of the International Association for Mathematical Geology*, 16(6):531–564.

Bedi, T., Zhu, B., Neugent, M. L., Lutz, K. C., De Nisco, N. J., and Li, Q. (2024). Bayesian modeling of co-occurrence microbial interaction networks. *arXiv preprint arXiv:2404.09194*.

Ewens, W. J. (1990). Population genetics theory-the past and the future. In *Mathematical and statistical developments of evolutionary theory*, pages 177–227. Springer.

Goodrich, J. K., Waters, J. L., Poole, A. C., Sutter, J. L., Koren, O., Blekhman, R., Beaumont, M., Van Treuren, W., Knight, R., Bell, J. T., et al. (2014). Human genetics shape the gut microbiome. *Cell*, 159(4):789–799.

Ishwaran, H. and James, L. F. (2001). Gibbs sampling methods for stick-breaking priors. *Journal of the American Statistical Association*, 96(453):161–173.

Neugent, M. L., Kumar, A., Hulyalkar, N. V., Lutz, K. C., Nguyen, V. H., Fuentes, J. L., Zhang, C., Nguyen, A., Sharon, B. M., Kuprasertkul, A., et al. (2022). Recurrent urinary tract infection and estrogen shape the taxonomic ecology and function of the postmenopausal urogenital microbiome. *Cell Reports Medicine*, 3(10).

Scher, J. U., Sczesnak, A., Longman, R. S., Segata, N., Ubeda, C., Bielski, C., Rostron, T., Cerundolo, V., Pamer, E. G., Abramson, S. B., et al. (2013). Expansion of intestinal prevotella copri correlates with enhanced susceptibility to arthritis. *elife*, 2:e01202.

Yoon, G., Gaynanova, I., and Müller, C. L. (2019). Microbial networks in SPRING-Semi-parametric rank-based correlation and partial correlation estimation for quantitative microbiome data. *Frontiers in Genetics*, 10:516.
